# An allometric model-based approach for estimating biomass in seven Indian bamboo species in western Himalayan foothills, India

**DOI:** 10.1038/s41598-022-11394-3

**Published:** 2022-05-09

**Authors:** R. Kaushal, S. Islam, Salil Tewari, J. M. S. Tomar, S. Thapliyal, M. Madhu, T. L. Trinh, Tarun Singh, Avnindra Singh, J. Durai

**Affiliations:** 1 ICAR-Indian Institute of Soil and Water Conservation, 218, Kaulagarh Road, Dehradun, 248 195 India; 2 G.B. Pant University of Agriculture and Technology, Pantnagar, India; 3 International Bamboo and Rattan Organization, Beijing, China

**Keywords:** Plant ecology, Forestry

## Abstract

The rapid growth rate, high biomass production, and annual harvesting make bamboo a suitable species for commercial production. Allometric equations for many broadleaf and conifer tree species are available. However, knowledge of biomass production and allometric equations of bamboos is limited. This study aims to develop species- specific allometric models for predicting biomass and synthetic height values as a proxy variable for seven bamboo species in Himalayan foothills. Two power form-based allometric models were used to predict aboveground and culm biomass using diameter at breast height (D) alone and D combined with culm height (H) as an independent variable. This study also extended to establishing an H–D allometric model that can be used to generate synthetic H values as a proxy to missing H. In the seven bamboo species studied, among three major biomass components (culm, branch and foliage), culm is the most important component with the highest share (69.56–78.71%). The distribution of percentage (%) share of culm, branch and foliage to above-ground fresh weight varies significantly between different bamboo species. *D. hamiltonii* has the highest productivity for above-ground biomass components. Ratio of dry to fresh weight of seven bamboo species was estimated for culm, branch, foliage and above-ground biomass to convert fresh weight to dry weight.

## Introduction

Bamboos are a group of perennial plants belonging to the Poaceae family. The rapid growth rate, high biomass production, and annual harvesting make bamboo a suitable species for commercial production. There are 1642 bamboo species which occupy a broad ecological regime across the globe, mainly in tropical and sub-tropical regions^1^. Worldwide, bamboo grows over 35 million ha area and covers 3.2 percent of the forest areas of their host countries or about 1 percent of the global forest area^2,3^. India is one of the richest countries globally in terms of bamboo genetic resources with 125 bamboo species belonging to 23 genera. The area under bamboo in the country is 15.68 million hectares which provide livelihood to about 2 million traditional artisans through harvesting, processing, value addition and selling bamboo products^4^. The demand for bamboo is estimated to be 26.69 million tonnes against the supply of 13.47 million tonnes in the country^5^. Overall, bamboo contributes to achieving important United Nations 2030 Agenda Sustainable Development Goals–particularly, SDG1, SDG7, SDG 11, SDG 12, SDG 13, SDG 15, and SDG 17^6^.

Climate change due to greenhouse gas emissions and fossil fuel resource depletion are major global concerns. India has set its target to reduce its emissions intensity by 33–35% between 2005 and 2030 and cut its carbon emissions to net-zero by 2070 at the United Nations Climate Change Conference (COP26) climate crisis summit 2021 organized in Glasgow, the UK. India is also committed to creating an additional carbon sink of 2.5 to 3 billion tonnes of CO_2_ equivalent through additional forest and tree cover by 2030. Climate change mitigation approaches broadly include, (i) conventional mitigation efforts (decarbonization technologies and techniques that reduce CO2), (ii) negative emissions techniques (bioenergy carbon capture and storage, biochar, enhanced weathering, direct air carbon capture and storage, ocean fertilization, ocean alkalinity enhancement, soil carbon sequestration, afforestation and reforestation, wetland construction and restoration) and (iii) radiative forcing geoengineering technologies (stratospheric aerosol injection, marine sky brightening, cirrus cloud thinning, space-based mirrors, surface-based brightening and various radiation management techniques)^7,8^. The radiative forcing geoengineering technologies are presently not included within policy frameworks^7^. So far, the IPCC assessments include two negative emissions technologies viz*.*, bioenergy carbon capture and storage and afforestation and reforestation to assess the feasibility of achieving the targets of the Paris agreement^9^.

Biomass-based techniques for CO_2_ removal include biomass for primary carbon storage (e.g., tree), combustion and subsequent storage of the products. Biomass produces biofuels as non-fossil fuel alternatives that supply 9% (~ 51 EJ) of the global overall primary energy requirement as an inexpensive, reliable, and sustainable energy source^10^. As a biofuel sources, biodiesel and biochar, can help mitigate the environmental effects of fossil fuels^11,12^. In this context, bamboo can also significantly reduce emissions by replacing fossil fuels for energy generation and sequestering carbon due to its capacity to grow quickly and persist for a long time without creating significant changes in the culm stock after harvesting, thereby helping to mitigate the effects of climate change^13–15^. Bamboo is a prospective and priority species for carbon storage and sequestration. Nath et al. (2015)^16^ reported that biomass carbon sequestration rates ranged from 13 to 24 Mg C ha^−1^ y^−1^ globally for various types of bamboo. Bamboos are considered equivalent to trees in afforestation and reforestation^17^ and have been certified under the verified carbon standard by the Food and Trees for Africa’s^18^. Bamboo has enormous potential to be used as feedstock for bioenergy production^19,20^. It can be used as an energy source by converting them into solid liquid and gaseous fuels. Compared to many non-food crops like miscanthus (*Miscanthus*), switchgrass (*Panicum virgatum*), giant reed (*Arundo donax*), short-rotation poplar coppices (*Populus*), or willow (*Salix*), bamboo can yield annually upto next life cycle of 30–50 years without disturbing the root system (roots and rhizome). This can additionally prevent soil erosion, improve soil carbon sequestration and lower the pressure on the existing forests. Littlewood et al. (2013)^21^ reported high sugar content of 62% of dry matter in two bamboo species from China. Despite vast potential of bamboo in producing biomass, bioenergy, carbon sequestration and providing numerous ecosystem services, bamboo has been neglected in many policy agreements related to climate change (United Nations Framework Convention on Climate Change (UNFCCC), the Kyoto Protocol and the Marrakech Accords) mainly due to its botanical classification as a grass^13,14,16,22^. Therefore, there is an urgent need to improve our understanding of biomass production and carbon sequestration by bamboos so that it can be recognized and included in the climate change programme of UNFCCC.

Assessment of woody biomass is helpful in timber extraction, tracking changes in the carbon stocks and quantifying the amount of carbon dioxide which can be sequestered from the atmosphere. Destructive methods and indirect methods (biomass equations) are generally used for estimating tree biomass. The use of biomass equations is cost-effective and less time-consuming. It is recognized by United Nations as a common framework and good practice guidance for carbon reporting in implementing the emerging carbon credit market mechanism^23^. Biomass equation predicts biomass based on easily correlated measured variables like diameter at breast height and tree height^24,25^. However, in bamboos biomass estimation becomes more complicated due to different aged and multiple culms. In bamboos, occular methods are being used for assessing biomass by the traders and farmers which require long field experience and suffer from a high degree of inaccuracy and therefore results in huge losses to the farmers, foresters, industries and other bamboo growers. Therefore, there is an urgent need to develop site-specific predictive models/ equations for estimating biomass in bamboos. Of various non-linear models, allometric models are most efficient for estimating biomass because of numerous computational advantages like high convergence of model parameters compared to most of the other non-linear models, best goodness of fit and the low statistical errors^26–29^.

In literature, allometric equations are available for broadleaf and conifer tree species. However, knowledge on biomass production in bamboos is limited. Most of the allometric equations for bamboo are available for *Phyllostachys* species in China^30–33^. In India, despite large area under bamboo, allometric equations are available for a few species viz., *Bambusa cacharensis, Bambusa balcooa, Bambusa tulda*, *Bambusa vulgaris, Dendrocalamus hamiltonii, Dendrocalamus longispathus, Melocanna baccifera, Schizostachyum dullooa* and *Pseudostachyum polymorphum* in north east India^34,35^; *D. strictus* in north India^36,37^; *Bambusa bambos* in eastern India^38^-and south India^39^. In north India, not much work has been attempted on biomass estimation in bamboos. The present study was therefore undertaken in seven commercial important bamboo species with objectives to (i) develop allometric models for seven bamboo species to predict both aboveground and culm biomass, (ii) establish height-diameter relationship for the bamboo species and, (iii) determine the biomass yield and relative contribution of different biomass components of this bamboo species.

## Materials and methods

### Study area

**Figure 1:**
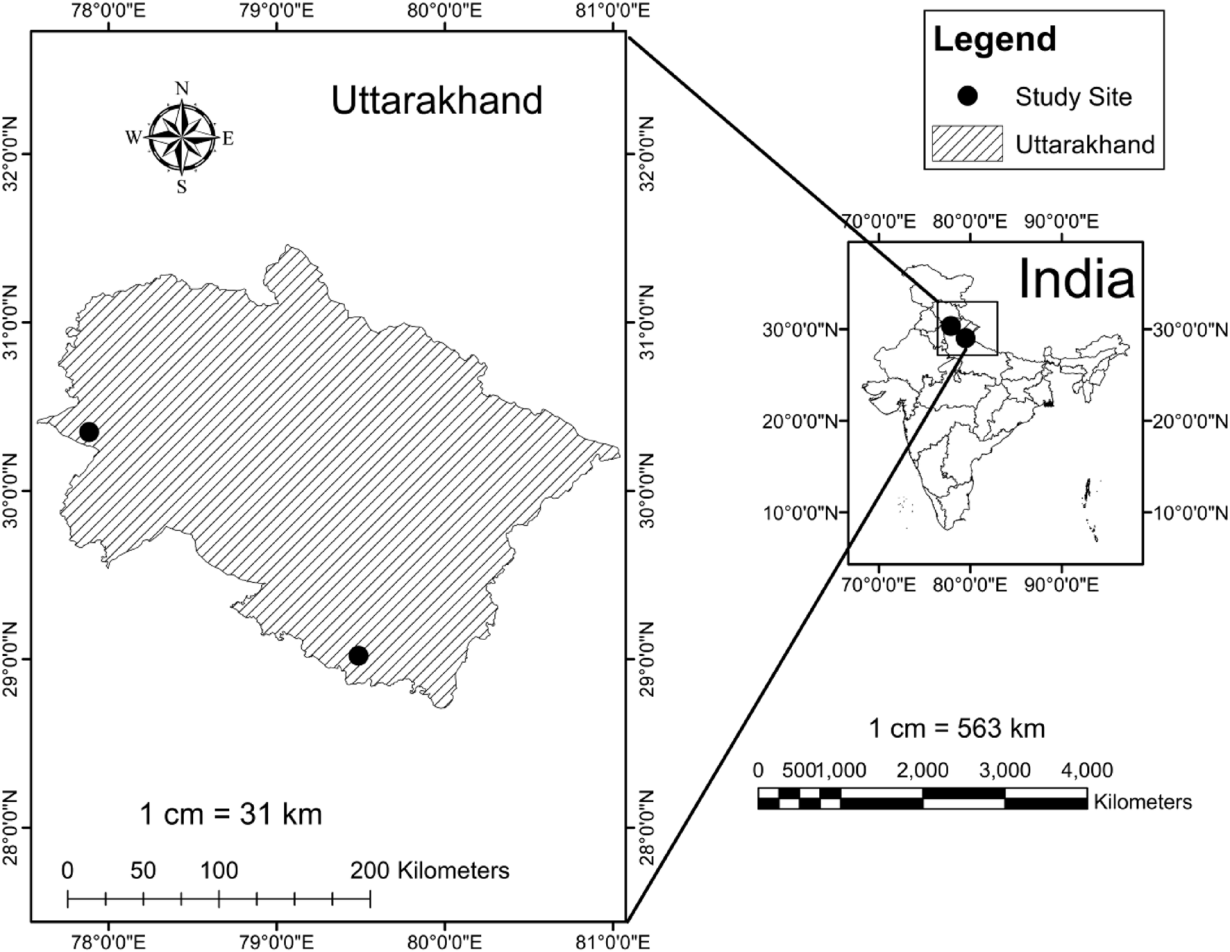
The study sites location map [Map was created using ArcGIS desktop (version 10.1) software].

The study was carried out in the western Himalayan foothills to develop allometric models for predicting biomass in seven commercial important bamboo species viz., *Bambusa balcooa, Bambusa bambos, Bambusa vulgaris, Bambusa nutans, Dendrocalamus hamiltonii, Dendrocalamus stocksii* and *Dendrocalamu strictus.* All the selected bamboo species are of commercial importance and on the priority list of the National Bamboo Mission, Government of India and International Bamboo and Rattan Organization (INBAR). Two sites located at a distance of 275 km were selected for collecting the data of seven different bamboo species (Fig. 1). The details of the study sites are given in Table 1. The planting material for site 1 was collected from the nursery of Uttarakhand Bamboo and Fiber Development Board, Dehradun, India. For site 2, the nursery raised plants were obtained from bamboo nurseries in different parts of the country. The identification of the bamboo species was based on morphological features described by Seethalakshmi and Kumar (1998)^40^.

**Table 1:** Detail of the study sites.

Parameters	Site1	Site 2
Location	Research farm, ICAR-Indian Institute of Soil and Water Conservation, Dhulkot and Selaqui	Agroforestry Research Centre, G.B. Pant University of Agriculture and Technology, Pantnagar
District/state/country	Dehradun, Uttarakhand, India	U.S. Nagar, Uttarakhand, India
Coordinates	30" 20′ 59" N latitude, 77" 53′ 05" E longitude	29° 1′ 17″ N latitude and 79° 29′ 14″ E longitude
Altitude	548 m	243.8
Soil type	Inceptisols	Mollisols
Climate	Sub-humid and subtropical climate	humid sub-tropical
Rainfall (mm)	1660	1364
Species	*Bambusa balcooa, B. bambos, B. vulgaris, B. nutans, Dendrocalamus hamiltonii, D. stocksii* and *D. strictus*	*Bambusa balcooa, Bambusa bambos, Bambusa nutans, Dendrocalamus hamiltonii,* and *Dendrocalamus strictus*
Year of plantation	2010 and 2 012	2005
Area of plantation	1.2 ha	3 ha

### Sampling

For sampling, three plots of 0.018 ha (15 m × 12 m) were selected for each species at site 1. For site 2, three plots of 0.0225 ha (15 m × 15 m) were selected for each species. Each plot consisted of 9 bamboo clumps comprising of numerous bamboo culms (poles). Sample selection was done using two-stage random sampling design considering clumps as 1st stage sampling unit and culms as 2^nd^ stage sampling unit. In total 39 plots (21 at site 1 and 18 at site 2) comprising 351 clumps were demarcated to collect data every year starting from 2017 to 2020. For understanding the culm size distributions within the sample plots, three clumps were selected randomly and measured for their clump girth, crown diameter, culm number, culm diameter at breast height. Depending on the culm sizes, different diameter classes were recognized representing the whole diameter range. From each diameter class, three mature culms (> 3 years old) were harvested randomly for each species in each clump. The age of mature culms in the clumps was determined on the basis of culm sheath, colour of culms, position of the culms, and growth and development of branches and leaves^41^. The summary statistics for biometric parameters of different bamboo species studied are given in Table 1.The diameter of each bamboo culm was measured at breast height with the help of a caliper. After harvesting the culm, its height was measured with the tape and separated into different components viz., leaf, branch and culm. Fresh weights were taken for respective components in the field. Subsamples from upper, middle, and lower portions of the culms of different ages were oven-dried at 65 °C to constant weight for determining the ratio of dry weight to the fresh weight^42^.

### Development of allometric equations for biomass estimation

Two power equation forms of allometric equations were developed for biomass modelling of seven bamboo species. The first one was a two-parameter general power form allometric equation as:

1 $$y_{i} = ax_{i}^{b} + \varepsilon_{i}$$

where ‘a’ denotes the constant of proportionality or scale constant, ‘b’ denotes the power or shape constant, and $\varepsilon_{i}$ is the error component following normal distribution with zero mean and non-constant variance $\sigma_{i}^{2}$ i.e.,$\varepsilon_{i} \sim iid\,N(0,\sigma_{i}^{2} )$. ‘x’ be a single predictor variable. Here, diameter at breast height (D) and culm volume (D^2^H) were used as x variables. Further, three-parameter power form of allometric equation was used as:

2 $$y_{i} = ax_{i}^{b} z_{i}^{c} + \varepsilon_{i}$$

where ‘a’ denotes the scale constant, ‘b’ and ‘c’ denote the shape constant for the predictor variable *x* and *z*, respectively; $\varepsilon_{i}$ be the error component follow a normal distribution with zero mean and non-constant variance $\sigma_{i}^{2}$ i.e.,$\varepsilon_{i} \sim iid\,N(0,\sigma_{i}^{2} )$. For biomass modelling *D* was used as x and *H* used as z.

The error term of both the allometric forms already discussed, assuming as heteroscedastic (i.e., not constant variance). Therefore, a weighted fit model was used to calculate the variance of the residual variation using the function,$\,\sigma_{i}^{2} = Var(\varepsilon_{i} ) = \hat{\sigma }^{2} (v_{i} )^{2\delta }$; where $\hat{\sigma }^{2}$ is the sum of the estimated errors of the squares; $v_{i}$ is the weighting variable associated with the *i*th plant/clump sampled; and $\delta$ is the coefficient of the variance function to be estimated^43–45^. Therefore, the weighted maximum likelihood non-linear fixed effects modelling method was used to fit the biomass equations for the seven bamboo species using the ‘nlme’ package of RStudio statistical software (R version 4.1.0). The details of the allometric form of equations used for testing and validation of best fit are shown in Table 2. Statistical differences between the biomass components of the seven species were examined using analysis of variance. Further post-hoc analysis was performed through Tukey’s Honest Significant Difference (HSD) test to achieve pair-wise comparison between species biomass components.

**Table 2:** Allometric models selected for comparison.

Model Id	Model name	Model Expression
A1	Allometric1	$y = aD^{b} + \varepsilon$
A2	Allometric2	$y = a(D^{2} H)^{b} + \varepsilon$
A3	Allometric3	$y = aD^{b} H^{c} + \varepsilon$

y denotes above ground fresh weight (kg), culm fresh weight (kg).

#### Model fit statistics and plot analysis to compare and choose the best model

Three popularly used models fit statistics viz. Akaike Information Criteria^46^, Bayesian Information Criteria^47^, and adjusted R^2^ (Adj.R^2^) were used to evaluate, compare and select the best allometric equations as per the following formulae:

(i) AIC = − 2ln(L) + 2p, where L is the likelihood of the model, and p is the number of model parameters and n is the sample size.
(ii) BIC = − 2ln(L) + pln(n), where L is the likelihood of the model, and p is the number of model parameters and n is the sample size.
(iii) $Adj.R^{2} = 1 - \frac{RSS/(n - p)}{{TSS/(n - 1)}}$, where RSS = Residual sum of square, TSS = Total sum of square, and p is the number of the parameters of the model and n is the sample size.

Model with the highest Adj.R^2^ and the lowest AIC, lowest BIC value was chosen as the best model^47^. Diagnostic plots of observed values versus fit and residual trends were also used to assess model performance.

### Model validation

Monte Carlo cross-validation method was applied to select, evaluate and compare the models^48–51^. The method divides the dataset randomly into two parts, with about 80% for model development and 20% for cross-validation, repeated R times (about 100 times) to obtain stable statistics. Model fitting statistics and cross-validation criteria were calculated for each data series randomly selected and averaged over R (approximately 100) series. To evaluate the predictive reliability of the fitted model, three statistical validation criteria viz., bias (percentage), Root Mean Squared Error (RMSE) and Mean Absolute Percentage Error (MAPE) was used^43–45,50–54^ as per the following formulae:.

$$ Bias(\% ) = \frac{1}{R}\sum\nolimits_{r = 1}^{R} \frac{100}{n} \sum\nolimits_{i = 1}^{n} {\frac{({y_{i} - \hat{y}_{i}) }}{{y_{i} }}} ,$$

$$ RMSE = \frac{1}{R}\sum\nolimits_{r = 1}^{R} {\sqrt {\sum\nolimits_{i = 1}^{n} {\left( {y_{i} - \hat{y}_{i} } \right)^{2} } } } {\text{ and}}$$

$MAPE(\% ) = \frac{1}{R}\sum\nolimits_{r = 1}^{R} \frac{100}{n} \sum\nolimits_{i = 1}^{n} {\frac{{\left| {y_{i} - \hat{y}_{i} } \right|}}{{y_{i} }}}$, where $y_{i}$ = Observed value and $\hat{y}_{i}$ = Predicated value, R = Number of times cross-validation occurred. A model with the lowest statistical error value in terms of bias, RMSE and MAPE were preferred as the best model.

### Assessment of the relationship between height and diameter of bamboo

Allometric equation of the form H = *aD*^*b*^ + *e*, was used to develop the relationship between height (H) with the diameter at breast height (D), where ‘a’ is the normalization (scale) constant, ‘b’ is the shape parameter and *e* is error term follows normal distribution with zero mean and non-constant variance $\sigma_{Hi}^{2}$ i.e., $e_{i} \sim iid\,N(0,\sigma_{Hi}^{2} )$. The model development was done using the weighted maximum likelihood nonlinear fixed effects modelling method and validation using Monte Carlo cross-validation method.

### Biomass and carbon stocks

Biomass in different components (culm, branches and foliage) at Site 1 was determined by harvesting three randomly selected culms of different aged clumps (from year 2013–19) for each species. Sub-samples from the upper, middle, and lower portions of the culms of different ages were oven-dried at 65 °C to constant weight to determine the dry weight to the fresh weight ratio which was used for determining biomass in different components^42^. The respective biomass values in different components were multiplied by the culm number and clump density for computing biomass on hectare basis and added to obtain total biomass for each species. Data on carbon stock and sequestration rate was estimated by assuming carbon concentration to 42%, 47% and 50% in foliage, branches and culm biomass respectively^34^. The carbon storage in the different culm components was determined by multiplying the biomass with the carbon concentration. The total carbon storage in the above ground standing biomass was obtained by summing the carbon concentration values for foliage, branch and culm components.

## Results

### Biometric parameters

**Figure 2:**
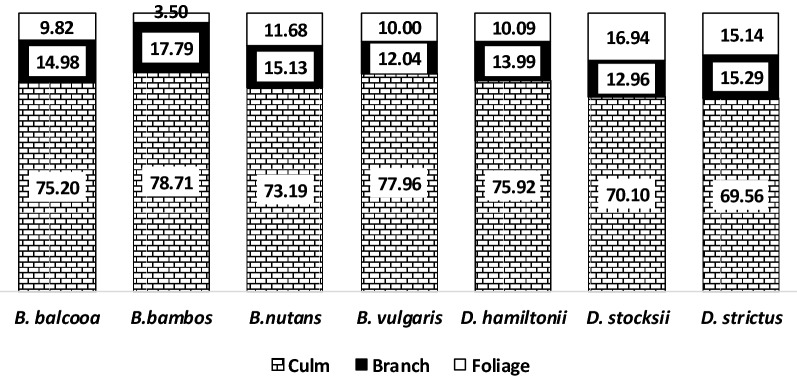
Distribution of percentage (%) share of culm, branch and foliage to above ground biomass in different bamboo species.

Analysis of variance revealed that different above-ground biometric parameters of the seven bamboo species viz., culm diameter, culm height, culm weight, foliage weight and above-ground biomass weight were varied significantly (*p* < 0.05). Post-hoc analysis through Tukey’s honest significant difference (HSD) test, revealed that *D. hamiltonii* has the highest average culm diameter (5.99 cm), culm height (11.91 m), culm weight (16.92 kg), foliage weight (1.53 kg) and above ground weight (20.39 kg). Average branch weight of *D. hamiltonii* though was observed as the highest (1.93 kg). Still it did not differ significantly from the other bamboo species viz., *B. balcooa, B. bambos, B. nutans* and *B. vulgaris* (Table 3). Furthermore, Tukey's HSD test demonstrated that for the seven bamboo species, the ratio of dry weight to fresh weight of distinct above-ground biomass components, such as culm, branch, foliage, and total aboveground biomass (AGB), differed considerably. aboveground *B. nutans* had the highest dry to fresh weight ratios for culm (0.63), branch (0.62), and AGB (0.61). *D. strictus*, on the other hand, had the highest dry to fresh weight ratio (0.53) for foliage (Table 4). The distribution of biomass revealed that the culm component has the largest percentage (69.56–78.71%), followed by branch (12.04–17.79%), and foliage (3.50–16.94%). *B. bambos* acquired the most branch biomass (17.79%) among the seven bamboo species, while *D. stocksii* accumulated the most culm (78.71%) and leaf biomass (16.94%) (Fig. 2).

**Table 3:** Summary statistics for different above ground biometric parameters.

Species (sample size)	Summary Statistics	Culm diameter (cm)	Culm height (m)	Culm weight (kg)	Branch weight (kg)	Foliage weight (kg)	Above ground weight (kg)
*B. balcooa* (97)	mean ± SE	5.18 ± 0.14^bc^	8.85 ± 0.33^c^	9.62 ± 0.68^b^	1.85 ± 0.12^a^	1.29 ± 0.09^bc^	12.76 ± 0.84^b^
	Min	2.55	3.30	0.70	0.10	0.05	1.03
	Max	8.6	17.2	26.00	4.86	2.99	32.88
*B. bambos* (54)	mean ± SE	5.37 ± 0.30^bc^	8.54 ± 0.55^c^	10.51 ± 1.40^b^	1.85 ± 0.15^a^	0.51 ± 0.08^d^	12.87 ± 1.65^b^
	Min	2.17	0.90	1.15	0.04	0.05	1.46
	Max	10.51	17.7	41.5	4.56	2.23	48.03
*B. nutans* (69)	mean ± SE	4.74 ± 0.16^ cd^	10.43 ± 0.41^b^	9.84 ± 0.88^b^	2.05 ± 0.28^a^	1.12 ± 0.06^c^	13.00 ± 1.14^b^
	Min	2.23	3.80	0.56	0.05	0.12	0.86
	Max	8.60	17.30	32.94	12.40	2.10	45.15
*B. vulgaris* (62)	mean ± SE	5.56 ± 0.18^ab^	9.39 ± 0.46^bc^	12.41 ± 0.95^b^	1.80 ± 0.17^a^	1.51 ± 0.11^ab^	15.71 ± 1.16^b^
	Min	2.77	2.7	0.9	0.08	0.04	1.15
	Max	8.60	18.80	31.58	5.895	3.545	38.34
*D. hamiltonii* (121)	mean ± SE	5.99 ± 0.19^a^	11.91 ± 0.43^a^	16.92 ± 1.17^a^	1.93 ± 0.07^a^	1.53 ± 0.07^a^	20.39 ± 1.29^a^
	Min	1.91	2.80	0.49	0.29	0.10	1.05
	Max	9.87	22.78	45.20	3.90	3.69	51.27
*D. stocksii* (83)	mean ± SE	4.24 ± 0.10^de^	8.61 ± 0.28^c^	4.97 ± 0.24^c^	0.95 ± 0.06^b^	1.20 ± 0.06^c^	7.12 ± 0.34^c^
	Min	1.91	1.60	0.72	0.10	0.05	1.20
	Max	5.73	13.30	10.15	2.25	3.20	14.60
*D. strictus* (176)	mean ± SE	3.78 ± 0.11^e^	6.24 ± 0.21^d^	3.96 ± 0.33^c^	0.84 ± 0.08^b^	0.59 ± 0.03^d^	5.38 ± 0.43^c^
	Min	1.30	1.50	0.06	0.03	0.03	0.17
	Max	8.60	13.5	28.27	6.73	1.95	34.17

SE: Standard Error of mean, Min.: Minimum value and Max.: Maximum value; mean ± SE value within biometric parameter, different superscript letters (a-e) in same column indicate significant differences (*p* < 0.05) between the species.

**Table 4:** Description about ratio of dry to fresh weight of different above ground biomass components.

Species	Ratio = W_sample dry weight_/W_sample wet weight_		
	Culm	Branch	Foliage
*B. balcooa*	0.59^a^	0.60^a^	0.48^d^
*B. bambos*	0.52^d^	0.49^d^	0.51^c^
*B. nutans*	0.53^c^	0.52^c^	0.46^e^
*B. vulgaris*	0.51^e^	0.57^b^	0.52^b^
*D. hamiltonii*	0.47^f^	0.49^e^	0.46^e^
*D. stocksii*	0.56^b^	0.45^f^	0.45^f^
*D. strictus*	0.53^c^	0.53^d^	0.53^a^

Dry weight (kg) = Ratio × Fresh weight (kg). Within biometric parameter, different superscript letters (a-f) in same column indicate significant differences (*p* < 0.05) between the species.

### Development and validation of allometric models

#### Aboveground biomass prediction

Using data obtained from harvested culms from > 3 years old bamboo plantations, three allometric equations [allometric form (1) in A1 and A2; allometric form (2) in A3] were established for the fresh aboveground biomass (AGB) and fresh culm weight for the seven bamboo species. Furthermore, utilising diameter at breast height (H) as a predictor of bamboo height (H), allometric model A1 was established once more. The ranging value of adjusted-R^2^ (Adj.R^2^) (0.82–0.97), AIC (190.24–562.79) and BIC (205.57–580.74) for the three models suggested differential fitting accuracy, but high Adj.R^2^ and low AIC and BIC value for the three model, evidenced appropriateness of adopting allometric models (Table 5).

Monte Carlo cross-validation method unfolded prediction accuracy of the fitted allometric models with the extended value of ABias (− 14.62 to 8.90%), ARMSE (0.98–5.12) and AMAPE (11.28–30.29%), revealed differential prediction accuracy of the fitted model. In contrast, low error values for the three models, indicating their prediction capabilities (Table 6).

Allometric model A1 unveiled the highest adj.R^2^ (0.94) for *B. bambos*, lowest AIC (209.02–269.03) and BIC (221.35–283.08) for *B. bambos* and *B. nutans.* Further, it also revealed the lowest prediction error for *B. bambos* and *B. nutans,* with the lowest ABias (− 2.74 to 0.50%) ARMSE (2.14–4.02) and AMAPE (10.83–25.88%)*.*With the lowest AMAPE (17.66–22.50%) and ARMSE (1.31–3.91)*,* the A2 model exhibited the lowest prediction error for *D.stocksii and B.vulgaris.* Furthermore, A2 found the lowest ABias (− 4.08%) for *B. vulgaris.*For *B. balcooa, D. hamiltonii* and *D. strictus,* model A3 revealed the highest adj.R^2^ (0.87–0.97), lowest AIC (335.17–550.55) and BIC (367.52–564.76)*.* Further, it also had the the lowest prediction error for *B. balcooa, D. hamiltonii and D. strictus,* with the lowest ABias (− 7.13 to − 0.47%) and AMAPE (18.96–23.45%) for *B. balcooa, D. hamiltonii, as well as* the lowest ARMSE (2.97–23.45) for *B. balcooa* and *D. hamiltonii*.

**Figure 3:**
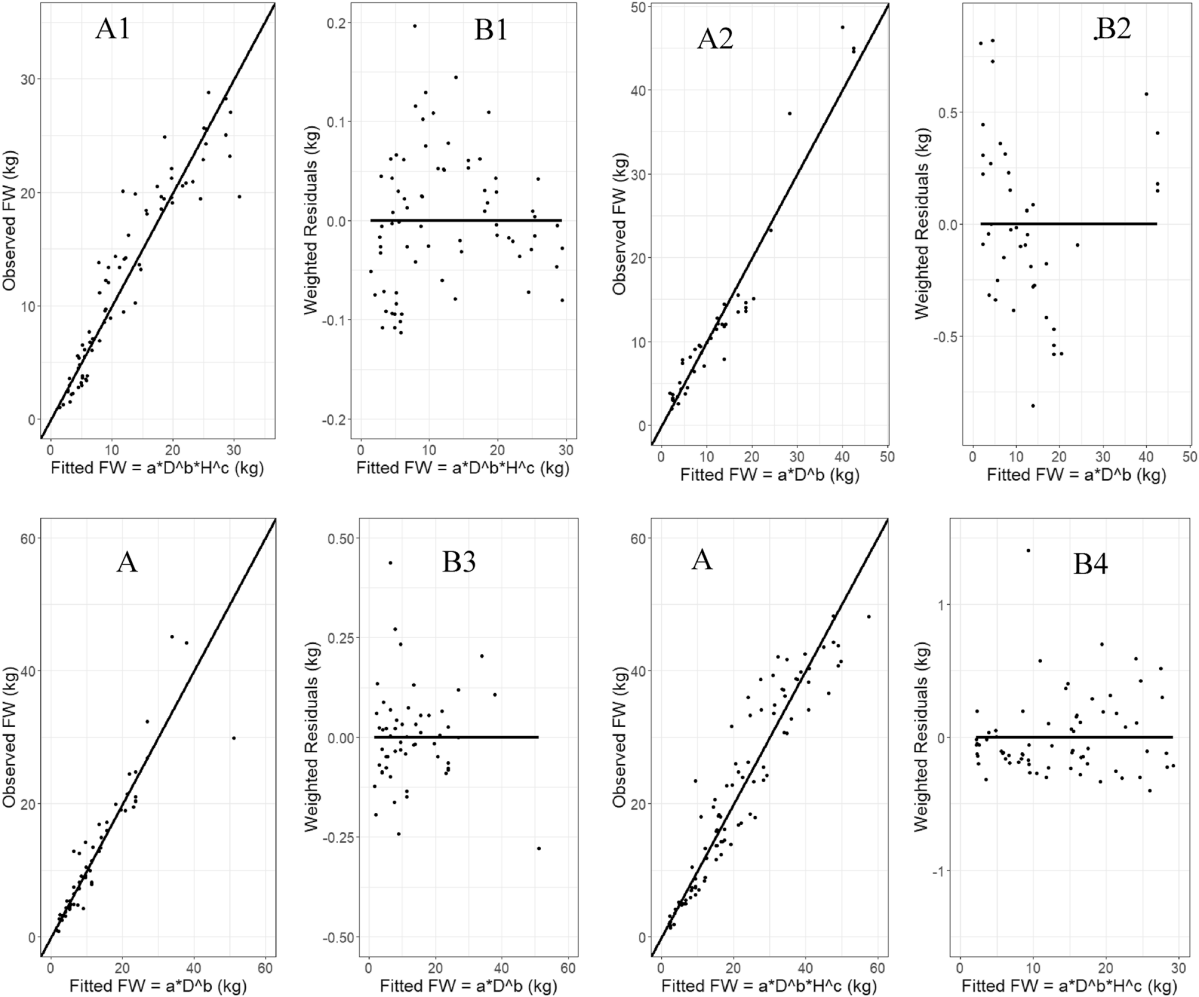
A1-A4 plots potraited observed vs best fitted allomteric models for above ground fresh weight prediction in *B. balcooa, B. bambos, B. nutans, D. hamiltonii, respectively*. Whereas B1-B4 plots potraited weighted residuals vs best fitted above ground fresh weight values in *B. balcooa, B. bambos, B. nutans, D. hamiltonii, respectively.*

**Figure 4:**
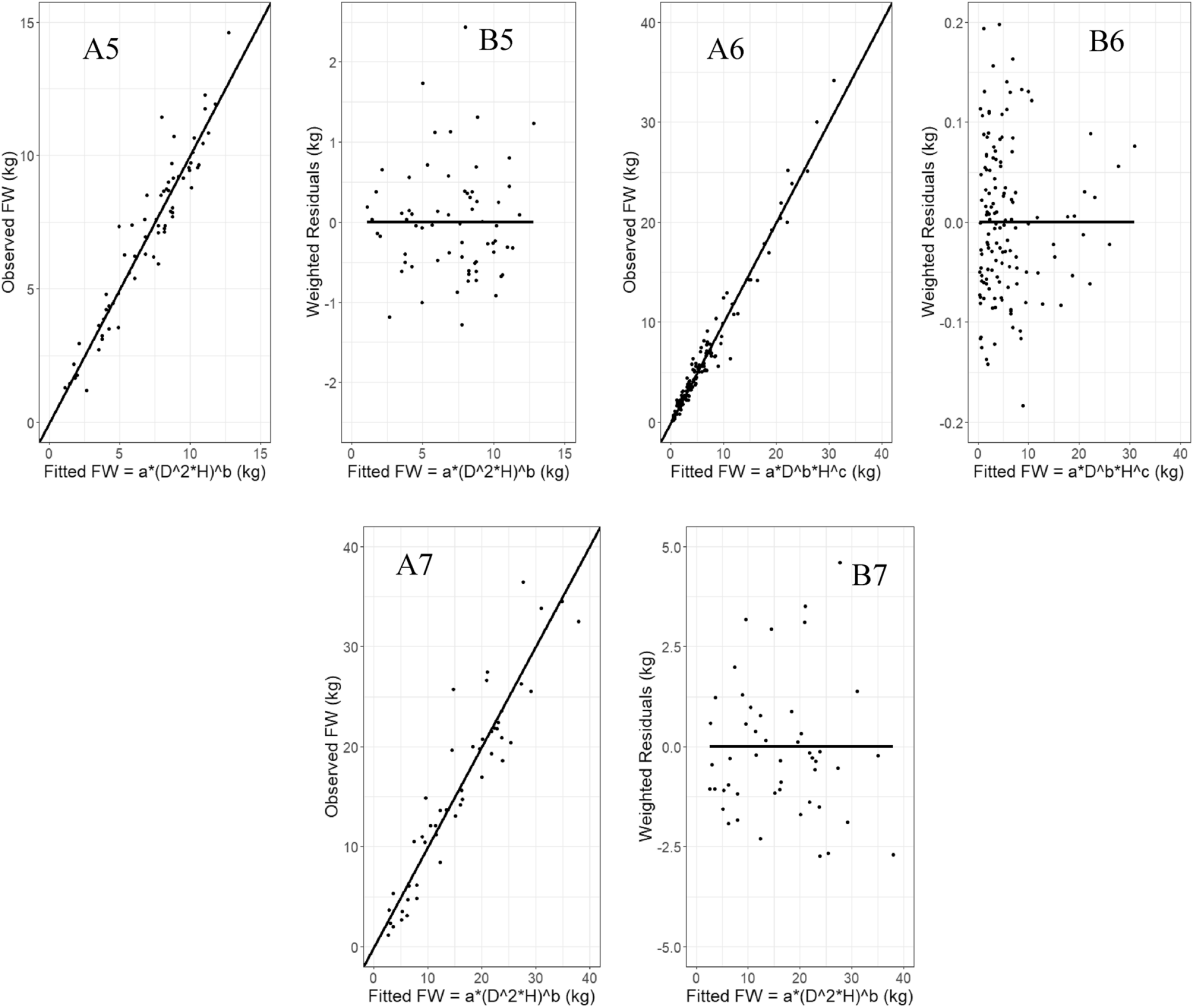
A5-A7 plots potraited observed vs best fitted allomteric models for above ground fresh weight prediction in *D. stocksii, D. strictus,* respectively. Whereas B5-B7 plots potraited weighted residuals vs best fitted above ground fresh weight values in *D. stocksii, D. strictus and B. vulgaris,* respectively*.*

Overall, the abovegroundA1 model was most efficient for *B.bambos* and *B.nutans*, whereas A2 was most reliable for *D.stocksii* and *B.vulgaris,* and A3 was most suitable for *B.balcooa, D.hamiltonii, and D.strictus,* according to the results of above ground biomass modelling (Tables 5 and 6). Figures 3 and 4 show the findings of the best fitted allometric models for above-ground fresh weight prediction. The results show that the weighted best fitted allometric models effectively capture heteroscedasticity in residuals and provide much lower residuals. Furthermore, residuals were found to be random (lack of any pattern) visually, indicating high fitting accuracy.

**Table 5:** Parameter estimates of allometric models fitted on 80% dataset for above ground fresh weight prediction.

Species	Model Id	Weight variable	Parameter [Estimate ± std. error]			Adj.R^2^	AIC	BIC
			a	b	c			
*B. balcooa*	A1	1/D^2.41^	0.10 ± 0.02	2.87 ± 0.11	–	0.85	375.02	391.42
	A2	1/D^0.40^	0.15 ± 0.03	0.79 ± 0.04	–	0.83	387.65	404.05
	A3	1/D^2.18^	0.10 ± 0.02	2.45 ± 0.19	0.31 ± 0.14	0.87	372.10	390.88
*B. bambos*	A1	1/D^1.10^	0.35 ± 0.06	2.04 ± 0.09	–	0.94	209.02	221.35
	A2	1/D^0.29^	0.24 ± 0.06	0.69 ± 0.04	–	0.92	225.08	237.40
	A3	1/D^1.16^	0.40 ± 0.06	2.34 ± 0.24	− 0.29 ± 0.20	0.94	215.00	234.37
*B. nutans*	A1	1/D^2.02^	0.21 ± 0.04	2.56 ± 0.11	–	0.82	269.03	283.08
	A2	1/D^0.34^	0.15 ± 0.04	0.80 ± 0.05	–	0.85	306.83	320.89
	A3	1/D^1.99^	0.16 ± 0.03	2.34 ± 0.17	0.27 ± 0.14	0.85	273.51	295.59
*D. hamiltonii*	A1	1/D^1.58^	0.29 ± 0.05	2.29 ± 0.08	–	0.87	562.79	580.74
	A2	1/D^0.58^	0.16 ± 0.03	0.77 ± 0.03	–	0.88	552.35	570.30
	A3	1/D^1.54^	0.20 ± 0.03	1.88 ± 0.13	0.43 ± 0.12	0.89	550.55	564.76
*D. stocksii*	A1	1/D^1.48^	0.37 ± 0.05	2.01 ± 0.9	–	0.86	208.4	223.72
	A2	1/D^0.23^	0.30 ± 0.04	0.62 ± 0.02	–	0.91	190.24	205.57
	A3	1/D^0.78^	0.29 ± 0.04	1.31 ± 0.16	0.58 ± 0.11	0.90	198.21	222.30
*D. strictus*	A1	1/D^1.78^	0.15 ± 0.01	2.48 ± 0.04	–	0.96	357.64	378.24
	A2	1/D^0.48^	0.10 ± 0.01	0.82 ± 0.01	–	0.97	347.55	368.14
	A3	1/D^1.75^	0.11 ± 0.01	1.92 ± 0.1	0.57 ± 0.09	0.97	335.17	367.52
*B. vulgaris*	A1	1/D^0.95^	0.30 ± 0.08	2.23 ± 0.15	–	0.84	275.31	288.56
	A2	1/D^0.32^	0.27 ± 0.07	0.70 ± 0.04	–	0.87	264.20	277.45
	A3	1/D^1.20^	0.26 ± 0.06	1.50 ± 0.21	0.63 ± 0.14	0.87	268.31	289.12

**Table 6:** Validation of allometric models of above ground fresh weight prediction on 20% dataset.

Species	Model Id	ABias (%)	ARMSE	AMAPE (%)
*B. balcooa*	A1	− 7.13	3.09	21.67
	A2	− 11.52	2.87	24.85
	A3	− 7.10	2.97	21.22
*B. bambos*	A1	− 2.74	4.02	25.88
	A2	2.77	4.17	27.95
	A3	− 14.62	5.12	30.29
*B. nutans*	A1	0.50	2.14	10.83
	A2	− 8.87	2.22	13.96
	A3	− 1.64	2.02	11.28
*D. hamiltonii*	A1	− 9.13	3.90	23.93
	A2	− 2.57	3.71	19.59
	A3	− 1.87	3.24	18.96
*D. stocksii*	A1	− 8.11	1.62	24.44
	A2	− 4.08	1.31	17.66
	A3	− 4.98	1.33	18.68
*D. strictus*	A1	− 3.98	0.98	24.22
	A2	− 0.65	1.12	27.28
	A3	− 0.47	1.03	23.45
*B. vulgaris*	A1	5.68	4.94	30.80
	A2	8.46	3.91	22.50
	A3	8.90	4.03	23.05

#### Culm biomass prediction

The establishment of allometric models for fresh culm biomass yielded lower Adj.R^2^ (0.80–0.96), AIC (200.39–554.83), and BIC (205.72–578.54) values than the fitted model for fresh above ground biomass, implying higher consistency of allometric models (Table 7). Cross-validation results of the fitted allometric models unfolded low prediction error with extended value of ABias (− 11.68 to 7.03%), ARMSE (0.79–4.70) and AMAPE (11.10–30.10%), revealing high prediction accuracy (Table 8).

**Table 7:** Parameter estimates of allometric models of culm fresh weight fitted on 80% dataset.

Species	Model Id	Weight variable	Parameter [Estimate ± std. error]			Adj.R^2^	AIC	BIC
			a	b	c			
*B. balcooa*	A1	1/D^2.91^	0.06 ± 0.01	3.00 ± 0.10	–	0.81	324.18	340.59
	A2	1/D^0.61^	0.07 ± 0.13	0.87 ± 0.03	–	0.89	311.20	337.61
	A3	1/D^2.67^	0.05 ± 0.01	2.33 ± 0.17	0.54 ± 0.12	0.85	315.14	340.92
*B. bambos*	A1	1/D^0.74^	0.16 ± 0.03	2.33 ± 0.10	–	0.95	200.39	212.72
	A2	1/D^0.22^	0.10 ± 0.03	0.79 ± 0.04	–	0.93	216.08	228.41
	A3	1/D^1.04^	0.25 ± 0.05	2.61 ± 0.25	− 0.39 ± 0.22	0.94	206.16	225.53
*B. nutans*	A1	1/D^2.28^	0.11 ± 0.02	2.75 ± 0.13	–	0.80	297.12	267.17
	A2	1/D^0.41^	0.10 ± 0.03	0.83 ± 0.06	–	0.81	291.67	205.72
	A3	1/D^1.68^	0.09 ± 0.02	2.11 ± 0.165	0.49 ± 0.15	0.80	550.33	578.54
*D. hamiltonii*	A1	1/D^1.77^	0.13 ± 0.03	2.59 ± 0.11	–	0.84	554.83	572.78
	A2	1/D^0.61^	0.07 ± 0.01	0.87 ± 0.03	–	0.88	545.05	563.01
	A3	1/D^1.68^	0.09 ± 0.02	2.12 ± 0.166	0.49 ± 0.14	0.87	550.33	578.54
*D. stocksii*	A1	1/D^1.35^	0.20 ± 0.06	2.31 ± 0.18	–	0.81	268.19	281.43
	A2	1/D^0.38^	0.18 ± 0.05	0.723 ± 0.04	–	0.84	253.26	265.50
	A3	1/D^1.39^	0.175 ± 0.05	1.37 ± 0.24	0.80 ± 0.165	0.84	258.48	279.28
*D. strictus*	A1	1/D^1.39^	0.087 ± 0.01	2.63 ± 0.05	–	0.95	287.58	308.17
	A2	1/D^0.43^	0.06 ± 0.01	0.86 ± 0.02	–	0.96	309.38	329.97
	A3	1/D^1.82^	0.07 ± 0.01	2.17 ± 0.11	0.46 ± 0.11	0.96	283.21	315.57
*B. vulgaris*	A1	1/D^1.35^	0.20 ± 0.06	2.31 ± 0.18	–	0.81	268.18	281.43
	A2	1/D^0.38^	0.18 ± 0.05	0.72 ± 0.04	–	0.84	253.26	266.50
	A3	1/D^1.38^	0.18 ± 0.05	1.37 ± 0.24	0.798 ± 0.16	0.83	258.47	279.28

**Table 8:** Validation of allometric models for culm fresh weight on 20% dataset.

Species	Model Id	ABias (%)	ARMSE	AMAPE (%)
*B. balcooa*	A1	− 9.71	2.96	25.23
	A2	− 7.52	2.56	20.38
	A3	− 8.69	2.71	21.98
*B. bambos*	A1	− 4.99	3.16	23.14
	A2	7.03	3.56	24.50
	A3	− 11.68	4.70	29.30
*B. nutans*	A1	2.27	1.76	12.91
	A2	− 2.05	1.71	11.10
	A3	− 3.03	1.79	18.86
*D. hamiltonii*	A1	− 7.08	4.16	26.07
	A2	− 8.24	3.93	23.98
	A3	− 11.28	3.57	24.56
*D. stocksii*	A1	7.01	4.70	27.66
	A2	6.11	3.87	21.46
	A3	6.92	3.89	22.44
*D. strictus*	A1	− 5.32	0.86	28.15
	A2	− 3.06	0.81	30.10
	A3	− 2.62	0.79	27.21
*B. vulgaris*	A1	7.01	4.70	29.66
	A2	6.11	3.77	22.46
	A3	6.92	3.83	25.44

Model A1 was found to be an outperformer for fresh culm biomass prediction of *B. bambos* as it has the highest fitting accuracy with adj.R^2^ (0.95), AIC (200.39) and BIC (212.72) and lowest prediction error with ABias (− 4.99%) ARMSE (3.16) and AMAPE (23.14%)*.* A2 model was found to be outperforming with maximum fitting and prediction accuracy over A1 and A3 model for five bamboo species viz., *B.balcooa, B.nutans, D.hamiltonii, D.stocksii and B.vulgaris.* Here, highest fitting accuracy of A2 was observed through the highest Adj.R^2^ (0.80–0.89), lowest AIC (253.26–545.05) and BIC (205.72–563.01). The highest prediction accuracy of A2 was defined by the lowest ABias (− 8.24 to 6.11%), ARMSE (1.71–3.93) and AMAPE (11.10–23.98%)*.*Whereas A3 was found to be most suitable for *D.strictus,* as it has the highest fitting accuracy [highest Adj.R^2^ (0.96), lowest AIC (283.21) and BIC (315.57)] and the lowest prediction error [ABias (− 2.62%) ARMSE (0.79) and AMAPE (27.21%)].

#### Generate synthetic height values as a proxy using H–D model

The results of fitted general power form allometric model for H–D modelling in the seven bamboo species revealed that the Adj.R^2^ value of the fitted model for the seven bamboo species was found to be > 0.7 except the species *B. nutans* with Adj.R^2^ = 0.69 (Table 9). *B. bambos*, on the other hand, had the highest Adj.R^2^ (0.89), lowest AIC (156.04) and BIC (497.83). In contrast, the highest AIC (497.83) and BIC (485.78) value were found in *D.hamiltonii.* Further, the prediction accuracy of the fitted models H–D was evaluated, and it was discovered that the bias of the fitted models was extended between − 1.57 (in *B. vulgaris*) and − 3.91 (in *D. stocksii*). The RMSE ranged between 1.16 (in *D. strictus*) to 2.70 (in *D. hamiltonii*). The lowest value of MAPE (9.34) was found in *B. bambos*, while the highest MAPE (20.52) was observed for *D. hamiltonii* (Table 10).

**Table 9:** Parameter estimates of H–D allometric model fitted on 80% dataset.

Species	Weight variable	Parameter [Estimate ± std. error]		Adj.R^2^	AIC	BIC
		a	b			
*B. balcooa*	1/D^0.91^	1.28 ± 0.19	1.17 ± 0.09	0.70	318.85	335.26
*B. bambos*	1/D^0.45^	1.29 ± 0.15	1.11 ± 0.06	0.89	156.04	168.37
*B. nutans*	1/D^1.07^	2.58 ± 0.37	0.89 ± 0.09	0.69	254	260.74
*D. hamiltonii*	1/D^0.38^	2.30 ± 0.30	0.92 ± 0.06	0.70	497.83	485.78
*D. stocksii*	1/D^0.41^	1.55 ± 0.21	1.18 ± 0.09	0.76	232.66	247.99
*D. strictus*	1/D^0.13^	1.73 ± 0.10	0.96 ± 0.03	0.84	438.77	459.36
*B. vulgaris*	1/D^1.057^	1.09 ± 0.22	1.24 ± 0.11	0.71	220.15	224.39

**Table 10:** Validation of H–D allometric model on 20% dataset.

Species	Bias (%)	RMSE	MAPE (%)
*B. balcooa*	− 3.33	1.61	13.73
*B. bambos*	− 2.26	1.25	9.34
*B. nutans*	2.14	1.47	12.80
*D. hamiltonii*	− 3.65	2.70	20.52
*D. stocksii*	− 3.91	1.58	12.61
*D. strictus*	− 1.72	1.16	16.66
*B. vulgaris*	− 1.57	1.48	10.67

### Biomass and carbon stocks

The biomass in different components revealed the maximum contribution by culms followed by branches and foliage except *B. vulgaris* where foliage biomass was more than branch biomass. The culm biomass followed the order: *B. vulagris* > *B. balcooa* > *B. nutans* > *D. hamiltonii* > *D. strictus* > *B. bambos* > *D. stocksii.* Branch biomass was highest in *B. nutans* followed by *B. balcooa* and minimum in *D. strictus,* while foliage biomass was highest in *B. nutans* followed by *B. vulgaris*. Total biomass was highest in *B. balcooa* (212.6 Mg ha^−1^), followed by *B. nutans* (209.2 Mg ha^−1^). The mean total biomass ranged from 11.87 Mg ha^−1^ y^−1^ in *D. stocksii* to 30.37 Mg ha^−1^ y^−1^ in *B. balcooa.* The biomass carbon storage in the present study ranged from 40.8–104.2 with annual carbon storage rate of 5.83–14.88 Mg ha^−1^ yr^−1^ (Table 11).

**Table 11:** Biomass and carbon storage in different above ground components in different bamboo species after 7 years of plantation.

Species	Culm (Mg ha^−1^)	Branch (Mg ha^−1^)	Foliage (Mg ha^−1^)	Total biomass (Mg ha^−^1)	Mean annual biomass (Mg ha^−1^ year^−1^)	Above ground carbon storage (Mg ha^−^1)	Mean carbon storage (Mg ha^−1^ year^−1^)
*B. balcooa*	171.6	23.0	18.0	212.6	30.37	104.2	14.88
*B. bambos*	70.9	15.5	2.9	89.2	12.75	43.9	6.27
*B. nutans*	160.3	30.0	18.9	209.2	29.89	102.2	14.60
*B. vulgaris*	174.4	15.7	18.4	208.4	29.77	102.3	14.61
*D. hamiltonii*	93.4	11.1	10.9	115.5	16.49	56.5	8.07
*D. strictus*	72.5	8.8	9.4	90.7	12.96	44.4	6.34
*D. stocksii*	64.6	14.8	3.6	83.1	11.87	40.8	5.83

## Discussion

### Biometric parameters of the seven bamboo species

In bamboo, culm has surprising biomass productivity due to its habit of producing sprouting from horizontal rhizome systems annually that allows annual harvesting without exposing the soil surface. Bamboo plays an important role in the global carbon cycle, by sequestering atmospheric carbon through its biomass accumulation process^55–58^. In the seven bamboo species studied, among three major biomass components (culm, branch and foliage), culm is the most important with the highest share (69.56–78.71%) to above-ground biomass which is more than double to combine biomass share of branch and foliage. Moreover, bamboo culm has high commercial value due to its multi-purpose utility^59^. The distribution of percentage (%) share of culm, branch and foliage to above-ground fresh weight varies significantly between different bamboo species (Fig. 2).

Analysis of variance (ANOVA) analysis revealed significant (*p* < 0.05) differential productivity of aboveground biometric components (culm diameter, culm height, culm fresh weight, foliage fresh weight and aboveground fresh weight) for the seven bamboo species. Tukey’s HSD post-hoc test, revealed that on an average *D. hamiltonii* has the highest biometric productivity for culm diameter D (5.99 cm), culm height H (11.91 m), culm fresh weight (16.92 kg), foliage fresh weight (1.53 kg) and aboveground fresh weight (20.39 kg). For branch fresh weight, though *D. hamiltonii* was found to have the highest productivity but not significantly higher than *B. balcooa, B. bambos, B. nutans* and *B. vulgaris* (Table 3). The differential growth rate of the seven bamboo species evidenced the need of species-specific relationship rather general relationship study for establishing the model for biomass prediction.

The ratio of dry to fresh weight of seven bamboo species in different components within species was found significantly different. The highest dry to fresh weight ratio was found for culm (0.63), branch (0.62) and above-ground biomass (0.61) in *B. nutans,* whereas, in foliage the highest ratio (0.53) was achieved for in *D. strictus*. The relationship equation between dry weight and fresh weight indicated that variability of fresh weight is directly proportional to dry weight (biomass) (Table 4).

### Development and validation of allometric models

Allometric relationship yields a non-destructive and indirect measurement of biomass components and is often the preferred approach since it is less time consuming and less expensive than direct measurements. Biomass production in the seven bamboo species has a high positive correlation with the diameter at breast height (D) and culm height (H) but did not have a significant correlation with age. This was similar to findings pointed out by Yen et al.^58^ for bamboo species *S. dullooa*, *P. polymorphum* and *M. baccifera*. So, D, combination of D and H was used for the prediction of biomass using combined data of more than three years old bamboo plantation. Many authors already observed D and D^2^H as good predictor variables for bamboo allometric modelling^59–61^. The results revealed that D was a valid independent variable but using volume (D^2^H) as compound independent variable or incorporation of H as a separate independent variable, further improved prediction accuracy for both above-ground biomass and culm biomass except bamboo species *B.bambos* and *B.nutans* for fresh above-ground biomass and *B.bambos* for fresh culm weight prediction (Tables 5, 6, 7 and 8). Here, the use of H as an additional independent variable did not improve prediction accuracy but increased prediction error may be due to measurement error in H data, or incorporation of H may have a masking effect on D during model fitting. A1 with D as independent variable alone, performed very well and was usually very close to A2 and A3 model. Further biomass equation using D as an independent variable has the practical advantage of easy measurement^61,62^. A1 model was also reported as a reliable model for biomass prediction by Yen et al.^41^, Kaushal et al.^36^

General power form allometric equation A2 with volume (D^2^H) as an independent variable, found to be most reliable for aboveground fresh weight prediction in bamboo species *D.stocksii* and *B.vulgaris*, whereas for fresh culm weight prediction A2 to be outperformer for five bamboo species viz.,* B.balcooa, B.nutans, D.hamiltonii, D.stocksii and B.vulgaris.*This finding was consistent with allometric models established for biomass prediction in thin-walled bamboo species by Singnaret al.^63^ and general findings of Chave et al.^53^ in woody species for aboveground biomass prediction using bole volume (D^2^H).

Three parameters allometric equation A3 with D and H as independent variables, were found to be most reliable in bamboo species *B. balcooa, D. hamiltonii* and *D. strictus* for aboveground fresh weight prediction, whereas for fresh culm weight prediction A3was most appropriate model in *D.strictus.* For A3 use of D and H as independent variables provided more flexibility to establish a relationship with biomass response.

The fitting and prediction accuracy of best fitted allometric models for fresh above weight prediction was visually investigated through line plots. The residual plot of prediction error values evidenced that allometric models using weighted maximum likelihood non-linear fixed effects modelling method capture heteroscedastic relationship between the biomass response and the predictor variables well and produced much lower residuals. Further, the residuals were found to be random (lack of any pattern) visually which indicates high fitting accuracy (Figs. 3 and 4).

Predictive models for biomass estimation provided the best goodness-of-fit statistics, increasing statistical efficiency in biomass estimate. The cross-validation procedure indicated that the parameter values in models are stable and therefore, the models are reliable to predict biomass. However, the models were data-driven, so growth relationship changes with alteration in climatic condition and the models developed are only valid within the range of DBH and height covered during sampling and also they do not account for other sources of variation in the study. Therefore, these models are only applicable to bamboo species with similar growth conditions.

### Generate synthetic values as a proxy height using H–D model

It was very often those best fitted allometric models were not possible to apply due to missing observation of H^64^. Synthetic H values were generated as proxy H using the H–D allometric model to get H value. H–D allometric equation developed for the seven bamboo species revealed high fitting accuracy with high Adj.R^2^ (> 0.7) except *B. nutans* (0.69). The highest fitting accuracy was observed in *B. bambos,* whereas the lowest value observed in *D. hamiltonii* (Tables 9 and 10). Similar, result was also pointed out by Kempeset al.^24^ and Sileshi^25^. The H–D relationship was developed using > 3-year-old bamboo plantation where H reached to plateau i.e., there is no growth in diameter and height^65^. As per our knowledge, for the first time, this study has established allometric modelling for quantifying H–D relationship between the seven-bamboo species viz., *B. bambos*, *B. nutans, D. stocksii*, *B. vulgaris, B. balcooa, D. hamiltonii, and D. strictus.*

### Biomass, carbon storage estimation and comparison

The biomass in different bamboo species ranged from 83.1 in *D. stocksii* to 212.6 in *B. balcooa*. Overall the values of *D.stocksii B. bambos*, *D. strictus*, and *D. hamiltonii* in the present study (83.1–115.5 Mg ha^−1^) are less than the values (206–212.6 Mg ha^−1^) of, *B. vulgaris, B. nutans* and *B. balcooa.* The difference in biomass is attributed to the differences in the culm size, culm height and culm density of the bamboo species and their differences in resource (water, light, nutrients, etc.) requirement and its utilization efficiency. Our values are within range to the reported average bamboo biomass of 124 Mg ha^−1^, with a range of 60–242 Mg ha^−1^ ^16^. Our values of biomass for *D. strictus* (90.7 Mg/ha for 7 years) are comparatively higher than the reported value of 11–36 Mg ha^−1^ for mature (5-year-old culms on > 20-year-old clump) dry tropical bamboo savannah with density of 253–267 ha^−1^ ^66^ and 30–49 Mg ha^−1^ in 3–5-year-old *D. strictus* plantation in a dry tropical region^67^. The values for *B. bambos* (89.23 Mg ha^−1^) is comparatively much lower than reported values of 109–167 Mg ha^−1^ for *B. bambos* plantation in eastern India^68^ and 287 Mg ha^−1^ for *B. bambos* in south India^69^. The lowest biomass in *B. bambos* is attributed to the large scale mortality of new bamboo shoots after the first harvest of clump in 2018. The values for *B. balcooa* and *B. vulgaris* 208 and 212 Mg ha^−1^ for 7 years with mean productivity of 29–30.3 Mg ha^−1^y^−1^ are higher than biomass produced from *Miscanthus* (5.9–13 Mg ha^−1^y^−1^)^70^ but comparable to the values of 121.5 Mg ha^−1^ for 4 years with mean productivity of 30.4 for mix stand of *B. cacharensis, B. vulgaris* and *B. balcooa* in north east India^71^.

The comparison of bamboo with other biomass producing species revealed that biomass production in *bamboo* species is comparable to many other short rotation woody tree species like *Leucaena leucocephala* (87 Mg ha^−1^) in 5 years); *Populus deltoides* (48.7–128 Mg ha^−1^ in 3–9-year-old plantation) and Eucalyptus (54.1–101.8 Mg ha^−1^ in 5 and 8-year-old plantations)^72–75^.

The carbon storage in the present study ranged from 40.8–104.2, which is also within reported values of mean carbon storage values of 30–121 Mg ha^−1^ ^16^. Our values are, however much lower than the carbon storage values of 169–259 Mg C ha^−1^ in bamboo forests in China and *Phyllostachys edulis* (169.4 Mg C ha^−1^), *Bambusa stenostachya* (114 Mg C ha^-1^) from Taiwan, *Bambusa bambos* (143.3 Mg C ha^−1^) from India^14^. Carbon storage values are, however comparable or greater than mean estimates of 39 and 86 Mg C ha^−1^, reported for forests in China and globally^76,77^. Compared to fast-growing short rotation trees, the carbon storage in bamboo species is much higher than the reported above ground carbon storage of 28.67 Mg ha^−1^ ^74^ and 22.04–23.23 Mg ha^−1^ in Poplar^78^.

The biomass carbon storage rate of 5.83–14.88 Mg ha^−1^ yr^−1^ in the present study is lower than 10-year-old *Dendrocalamus latiflorus* (16 Mg ha^−1^y^−1^)^79^ but within the reported range of 6–24 Mg C ha^−1^ yr^−1^ for various types of bamboo worldwide^16^. Compared to other woody tree species, carbon storage rate in the present study is higher than *Grewia optiva* (0.63–0.81 Mg ha^−1^y^−1^)^26^ and different agroforestry practices (0*.*29–15*.*21 Mg ha^−1^ yr^−1^)^80^. The difference in carbon sequestration potential may be ascribed to difference in tree density, age, structure and carbon concentration in different components^26,74^.

## Conclusions

In this study species-specific, two-parameter allometric equations with simple and compound regressor variable and three-parameter allometric equations were developed for aboveground biomass. Further, species-specific height-diameter models were also developed to predict bamboo height, which can be used as a proxy of height variable and used for allometric equations without measuring the height of bamboo. Since the allometric equations were developed using rigorous criteria, we can also conclude that the developed biomass estimation equations provide better biomass prediction for culm and above-ground biomass. The models can be expected to increase accuracy in estimation of biomass and carbon sequestration in bamboo-based ecosystems of India under managed natural stands or plantations grown under similar conditions. Moreover, considering the wide multipurpose use of the bamboo species, the developed models may provide useful information about culm biomass and above-ground biomass to forestry professionals, bamboo farmers, and other stakeholders.

### Limitations

Age-wise modelling and root biomass was not incorporated in this study due to some methodological difficulties. Inclusion of these parameters in the future can improve the prediction accuracy. Further, these models were derived from a limited data set covering Himalayan foothills which restricts its applicability to similar site conditions.

### Ethics approval

The planting material was obtained from different Government nurseries from different part of the country and for which all the permissions were obtained. All the collection complies with relevant institutional, national, and international guidelines and legislation.

## Data Availability

The datasets used and/or analysed during the current study are available from the corresponding author on reasonable request.
